# Longitudinal evolution of cortical thickness signature reflecting Lewy body dementia in isolated REM sleep behavior disorder: a prospective cohort study

**DOI:** 10.1186/s40035-023-00356-y

**Published:** 2023-05-22

**Authors:** Jung Hwan Shin, Heejung Kim, Yu Kyeong Kim, Eun Jin Yoon, Hyunwoo Nam, Beomseok Jeon, Jee-Young Lee

**Affiliations:** 1 Department of Neurology, Seoul Metropolitan Government-Seoul National University Boramae Medical Center and Seoul National University College of Medicine, Seoul, South Korea; 2 Department of Nuclear Medicine, Seoul Metropolitan Government-Seoul National University Boramae Medical Center and Seoul National University College of Medicine, Seoul, South Korea; 3 Institute of Radiation Medicine, Medical Research Center, Seoul National University, Seoul, South Korea; 4 Department of Neurology, Seoul National University Hospital and Seoul National University College of Medicine, Seoul, South Korea

**Keywords:** Rapid eye movement sleep behavior disorder, Dementia with Lewy bodies, Lewy body disease, Cortical thickness, Spatial covariance pattern, Magnetic resonance imaging

## Abstract

**Background:**

The isolated rapid-eye-movement sleep behavior disorder (iRBD) is a prodromal condition of Lewy body disease including Parkinson's disease and dementia with Lewy bodies (DLB). We aim to investigate the longitudinal evolution of DLB-related cortical thickness signature in a prospective iRBD cohort and evaluate the possible predictive value of the cortical signature index in predicting dementia-first phenoconversion in individuals with iRBD.

**Methods:**

We enrolled 22 DLB patients, 44 healthy controls, and 50 video polysomnography-proven iRBD patients. Participants underwent 3-T magnetic resonance imaging (MRI) and clinical/neuropsychological evaluations. We characterized DLB-related whole-brain cortical thickness spatial covariance pattern (DLB-pattern) using scaled subprofile model of principal components analysis that best differentiated DLB patients from age-matched controls. We analyzed clinical and neuropsychological correlates of the DLB-pattern expression scores and the mean values of the whole-brain cortical thickness in DLB and iRBD patients. With repeated MRI data during the follow-up in our prospective iRBD cohort, we investigated the longitudinal evolution of the cortical thickness signature toward Lewy body dementia. Finally, we analyzed the potential predictive value of cortical thickness signature as a biomarker of phenoconversion in iRBD cohort.

**Results:**

The DLB-pattern was characterized by thinning of the temporal, orbitofrontal, and insular cortices and relative preservation of the precentral and inferior parietal cortices. The DLB-pattern expression scores correlated with attentional and frontal executive dysfunction (Trail Making Test-A and B: *R* = − 0.55, *P* = 0.024 and *R* = − 0.56, *P* = 0.036, respectively) as well as visuospatial impairment (Rey-figure copy test: *R* = − 0.54, *P* = 0.0047). The longitudinal trajectory of DLB-pattern revealed an increasing pattern above the cut-off in the dementia-first phenoconverters (Pearson’s correlation, *R* = 0.74, *P* = 6.8 × 10^−4^) but no significant change in parkinsonism-first phenoconverters (*R* = 0.0063, *P* = 0.98). The mean value of the whole-brain cortical thickness predicted phenoconversion in iRBD patients with hazard ratio of 9.33 [1.16–74.12]. The increase in DLB-pattern expression score discriminated dementia-first from parkinsonism-first phenoconversions with 88.2% accuracy.

**Conclusion:**

Cortical thickness signature can effectively reflect the longitudinal evolution of Lewy body dementia in the iRBD population. Replication studies would further validate the utility of this imaging marker in iRBD.

**Supplementary Information:**

The online version contains supplementary material available at 10.1186/s40035-023-00356-y.

## Introduction

Isolated rapid eye movement (REM) sleep behavior disorder (iRBD) is associated with synucleinopathy and in most cases, evolves into Lewy body disease (LBD) [1, 2]. Dementia-first (dementia with Lewy bodies, DLB) and parkinsonism-first (Parkinson’s disease, PD) phenoconversion are major waypoints in the progression of iRBD patients [1, 3], for which clinical and pathological characteristics of the two conditions are distinct. The differences in prodromal conditions may be responsible for the heterogeneity in the clinical, neuropsychological and neuroimaging characteristics of iRBD [1, 4–6]. To efficiently monitor disease progression, it would be useful to stratify heterogeneous iRBD patients into homogenous subpopulations who will convert to dementia-first or parkinsonism-first LBD. However, an objective biomarker that can easily and reliably predict subtype-specific phenoconversion in iRBD has not been clearly established.

DLB patients have progressive cognitive decline with characteristic cognitive fluctuations, vivid hallucination, parkinsonism and RBD as core clinical features [7]. Significant cortical thinning in the temporoparietal, insula, cingulate, orbitofrontal and lateral occipital cortices has been characterized in DLB [8, 9]. Cortical thinning has also been reported in frontal, parietal, temporal, cingulate and occipital cortices in iRBD patients [4, 10–12]. Notably, cortical thinning is more profound in iRBD patients with mild cognitive impairment (MCI) than in iRBD patients without MCI [4], and the percentage of MCI is significantly higher in dementia-first converters than in parkinsonism-first converters [1]. Furthermore, a recent study reported that a low value of mean cortical thickness may predict overall phenoconversion in iRBD [13]. However, a multidimensional approach using a disease-related cortical thinning pattern and mean cortical thickness that reflects the longitudinal evolution of dementia-first LBD has not been studied thus far.

To develop an imaging biomarker predicting Lewy body dementia in an iRBD population, we set out to characterize magnetic resonance imaging (MRI)-driven whole-brain cortical signature from DLB and apply it in our prospective iRBD cohort. With longitudinal data, we analyzed the evolution of the cortical signature and the relationship between the imaging marker change and clinical phenoconversion in individuals with iRBD.

## Methods

### Participants

This study included healthy controls, iRBD patients, DLB patients, and Alzheimer’s dementia (AD) patients as a disease control. Participants were recruited from the neurology clinic at the Seoul National University Boramae Medical Center between 2013 and 2020. Healthy controls and iRBD patients were part of the age-matched prospective cohort reported in our previous studies [14, 15]. iRBD patients were diagnosed based on the International Classification of Sleep Disorders, 3rd edition [16] with video polysomnography confirmation. Healthy controls who visited our clinic for routine health checkups were prospectively enrolled. The DLB patients were diagnosed with probable DLB according to the 4th consensus criteria [7] with a clinical history of RBD as a suggestive feature. All AD patients were diagnosed according to the National Institute on Aging-Alzheimer’s Association Criteria [17] supported by positive (≥ grade-2 positivity) amyloid deposition with ^18^F-florbetaben PET [18, 19] performed within 2 years from the time of enrollment. We enrolled early AD patients with disease duration less than 5 years from symptom onset and a clinical dementia rating (CDR) of 0.5 or 1. All participants in the prospective cohort had given written informed consent, and the Institutional Review Board of Boramae Medical Center approved this study (30-2021-18).

### Clinical evaluation

We collected baseline demographic and clinical information including RBD symptom onset and disease duration. Olfactory function was tested with the Brief Smell Identification Test. The patients were evaluated on the Movement Disorders Society revised-Unified PD Rating Scale (MDS-UPDRS). Cognitive status was evaluated with the Mini-Mental Status Exam (MMSE), digit-span test, Korean color word Stroop Test, Trail Making Test (TMT)-A and -B, Controlled Oral Word Association Test, Seoul Verbal Learning Test (SVLT), Rey Complex Figure Test (RCFT) copy and Korean Boston naming test. In a subpopulation of iRBD patients (*n* = 31), imaging and clinical biomarkers were longitudinally followed up at 2 and 4 years from baseline. We followed up iRBD participants at the outpatient clinic every 3–4 months after enrollment and phenoconversion was assessed at every visit. We defined ‘phenoconverter’ as an iRBD patient developing clinical symptoms and signs fulfilling the diagnostic criteria of DLB [7], PD [20] or multiple system atrophy (MSA) [21] during the follow-up.

### Image acquisition and cortical thickness analyses

All participants underwent 3-T MRI at baseline. MRI data were acquired using a Philips Achieva 3-T MRI scanner (Philips Medical Systems, Netherlands) with a standard whole-head coil. A high-resolution 3D T1-weighted MRI sequence was obtained covering the whole brain for anatomical reference (224 slices, TR = 9.9 ms, TE = 4.6 ms, slice thickness = 1 mm, flip angle = 8°, FOV = 220 × 220 mm^2^, voxel size = 1 × 1 × 1 mm^3^). Cortical thickness analysis was performed by surface-based morphometry using Computational Anatomy Toolbox (CAT12) (http://www.neuro.unijena.de/cat) embedded in SPM12 (http://www.fil.ion.ucl.ac.uk/spm) that provides a fully automated method to estimate cortical thickness. All images underwent automated segmentation to gray matter, white matter, and cerebrospinal fluid; affine registration to an MNI template space; and then a nonlinear deformation. Finally, all images were smoothed with a 12-mm FWHM of Gaussian smoothing kernel and then resampled to 32 k template space. Quality of resampled surface data was assessed using the module provided by the CAT tool. We extracted cortical thickness of 74 regions of interest (ROIs) in each hemisphere defined by the Destrieux atlas [22] using CAT. The cortical surface was resampled onto the average subject and smoothed with a 10-mm, full-width half-maximum Gaussian kernel. The scaled subprofile model of principal component analysis (PCA) of the cortical thickness data was performed in the following steps using custom-written code with MATLAB 2019b (MathWorks, Natick, MA) [23]. From the cortical thickness matrix of DLB (*n* = 22, Table 1) and age-matched control samples (*n* = 17; age: 74.41 ± 2.53, male/female: 6/11), we derived a covariance matrix for cortical thickness in every ROI. Subsequently, we designed multiple linear regression models using every combination of the first 5 principal components (PCs) as independent variables and ‘group’ (DLB versus healthy control) as a dependent variable. We computed Akaike information criterion (AIC) in models with every combination of PCs for differentiation of DLB from healthy control. The model with the lowest AIC value was designated as the "DLB-pattern". The weights from all ROIs contributed to the DLB-pattern score [24]. To identify the stable regions among the ROIs, the weights of each ROI from DLB patients and healthy controls were boot-strap resampled 10,000 times (Additional file 1: Fig. S1). The Alzheimer’s disease-related cortical thickness covariance pattern (AD-pattern) was derived with the same methods that were applied to derive the DLB-pattern. We rechecked the lack of a significant influence of age by performing correlation analyses of age with DLB-pattern (*R* = − 0.2, *P* = 0.18) and AD-pattern (*R* = 0.02, *P* = 0.89) in healthy controls. In addition, the mean value of whole-brain cortical thickness in each subject was defined as the average thickness of 148 ROIs in both hemispheres. The mean cortical thickness was negatively correlated with age (*R* = − 0.30, *P* = 0.044). We calculated z-scores for the expression of the DLB-pattern, AD-pattern and the mean cortical thickness in each subject based on the mean and standard deviation of healthy controls.

**Table 1:** Baseline clinical characteristics of the participants in this study

	Healthy controls	iRBD	DLB	*P* value
*n*	44	50	22	
Male/female	15/29	27/23	13/8	0.14
Age (years)	68.6 ± 6.3	70.6 ± 5.9	76.1 ± 5.9	6.37 × 10^–5a^
RBD duration (years)	–	5.0 ± 4.7	–	0.47
MMSE	27.7 ± 2.1	27.0 ± 2.6	18.7 ± 5.3	2.62 × 10^–9b^
B-SIT	–	6.2 ± 2.8	–	0.84
MDS-UPDRS part I	–	8.1 ± 5.8	15.8 ± 10.1	0.0032
MDS-UPDRS part II	–	4.1 ± 4.2	15.4 ± 11.3	1.02 × 10^–5^
MDS-UPDRS part III	–	7.2 ± 6.0	39.3 ± 18.2	6.67 × 10^–11^
Hoehn & Yahr stage	–	–	2.3 ± 0.7	–
Digit span test	− 0.48 ± 0.68	0.11 ± 0.90	− 0.29 ± 1.41	0.14
TMT-A	− 0.097 ± 0.39	− 0.32 ± 1.01	− 1.22 ± 1.99	0.29
CWST	− 0.21 ± 0.90	− 0.77 ± 1.22	− 1.45 ± 1.29	0.092
BNT	0.67 ± 0.45	− 0.16 ± 1.17	− 1.60 ± 0.89	1.50 × 10^–6c^
RCFT	− 0.98 ± 1.16	− 1.44 ± 1.52	− 2.49 ± 1.47	0.016^d^
SVLTi	0.43 ± 1.02	− 0.31 ± 0.97	− 1.25 ± 0.86	2.00 × 10^–4e^
SVLTd	0.37 ± 0.93	− 0.62 ± 1.12	− 1.33 ± 1.16	8.33 × 10^–4f^
SVLTr	0.77 ± 0.68	− 0.21 ± 1.14	− 1.25 ± 1.24	6.93 × 10^–5g^
COWAT	− 0.083 ± 0.81	− 0.56 ± 1.01	− 1.07 ± 1.65	0.064
TMT-B	0.54 ± 0.20	− 1.49 ± 2.47	− 4.00 ± 2.66	3.12 × 10^–4h^

All scores are shown as the mean ± SD. Wilcoxon’s rank-sum test was used to compare MDS-UPDRS part I, II and III between iRBD and DLB. Kruskal–Wallis test and chi-square test were used to compare age and sex distribution and clinical profiles between healthy controls, iRBD, and DLB. *Post-hoc* Dunn’s test was used to compare group differences as follows

^a^DLB vs healthy controls (*P* = 3.70 × 10^–5^), DLB vs iRBD (*P* = 0.0024)

^b^DLB vs healthy controls (*P* = 2.00 × 10^–8^), DLB vs iRBD (*P* = 1.84 × 10^–7^)

^c^DLB vs healthy controls (*P* = 6.29 × 10^–6^), DLB vs iRBD (*P* = 9.55 × 10^–5^)

^d^DLB vs healthy controls (*P* = 0.039), DLB vs iRBD (*P* = 0.032)

^e^DLB vs healthy controls (*P* = 3.37 × 10^–4^) and DLB vs iRBD (*P* = 0.0046)

^f^DLB vs healthy controls (*P* = 6.56 × 10^–4^) and DLB vs iRBD (*P* = 0.038)

^g^DLB vs healthy controls (*P* = 6.03 × 10^–5^), DLB vs iRBD (*P* = 0.0092) and healthy control vs iRBD (*P* = 0.033)

^h^DLB vs healthy controls (*P* = 2.34 × 10^–4^) and healthy control vs iRBD (*P* = 0.011)

*RBD* rapid-eye-movement sleep behavior disorder, *DLB* dementia with Lewy bodies, *MMSE* Mini-mental status exam, *B-SIT* Brief smell identification test, *MDS-UPDRS* Movement Disorders Society-Unified Parkinson Disease Rating Scale, *BNT* Boston Naming Test, *COWAT* Controlled Oral Word Association Test, *CWST* Korean Color Word Stroop Test, *RCFT* Rey Complex Figure Test copy, *SVLT* Seoul Verbal Learning Test, *TMT* Trail Making Test

### Dopamine transporter (DAT) imaging acquisition

All patients with iRBD underwent an ^18^F-FP-CIT PET scan at baseline (Philips Gemini TF-64 PET/CT scanner, Philips Healthcare, Best, Netherlands). The PET scans (10-min emssion) were performed 2 h after intravenous injection of 185 MBq of ^18^F-FP-CIT. Prior to the PET scan, all iRBD patients were confirmed to be either in a drug-naive state or a medication-off state for at least 12 h. Detailed imaging protocols have been described in a previous study [14].

### Statistical analyses

Global cortical thickness was compared between the DLB and HC groups by a two-sample *t*-test using second-level models and algorithms provided by the CAT12 software package (r1733) within SPM12 (r7771) while controlling for age. The significance level was set at *P* < 0.01 and family-wise error correction was applied. We used Kruskal–Wallis test with Dunn’s *post-hoc* test and Wilcoxon’s rank-sum tests for statistical comparisons of the means. The correlation between clinical parameters and the cortical thickness signature was performed with partial correlation with age and sex as cofactors. For the correlation analyses between the cortical thickness signature and cognitive profiles, age, sex, and education year were included as cofactors. Cox-proportional hazards analyses adjusted for baseline age and sex were used to calculate the hazard ratio (HR). Kaplan–Meier survival analyses were used to draw survival curves related to each imaging marker. The sensitivity, specificity and diagnostic accuracy were calculated for detecting dementia-first phenoconversion among all disease converters. All statistical analyses were performed using custom-written code in MATLAB 2019b (MathWorks, Natick, MA).

## Results

### Baseline clinical characteristics of the enrolled population

**Fig. 1:**
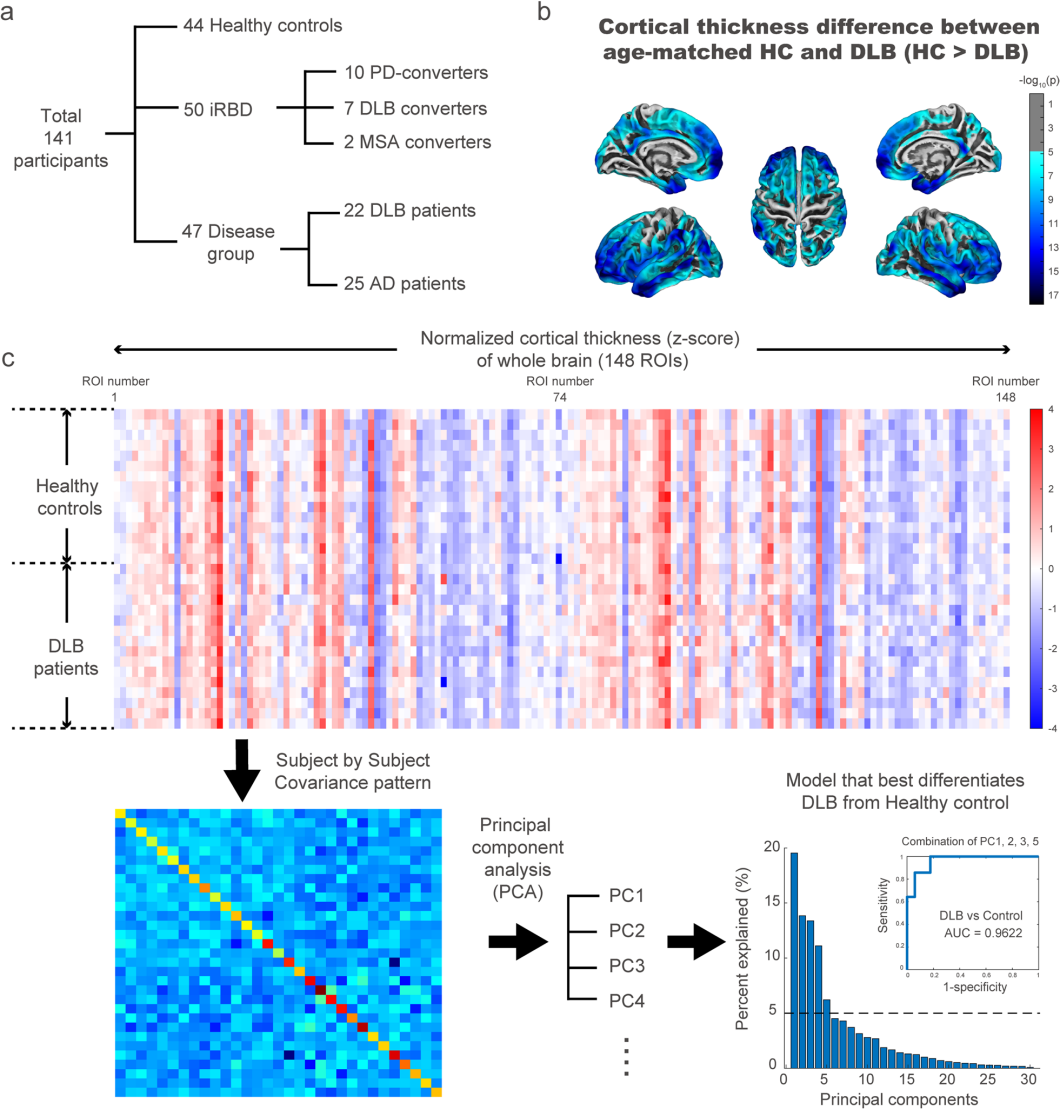
Enrolled population in this study and derivation of DLB-pattern. **a** A flow chart of enrolled participants in this study. **b** Cortical thinning in the DLB patients compared with healthy controls showed significant thinning in bilateral temporal, frontal, parietal, and occipital cortices (corrected *P* < 0.01). **c** Schematic figure showing the derivation of the DLB-pattern. The normalized cortical thickness matrix was collected in DLB patients and healthy controls followed by the derivation of the subject by subject covariance pattern. The colored boxes corresponding to each ROI of cortical thickness were assigned a label ranging from 1 to 148, as listed in Additional file 1: Table S1. The covariance pattern was fed into the PCA analyses which resulted in principal components of cortical thickness spatial pattern. The best model that differentiated DLB patients from healthy control was chosen and the normalized weights for all ROIs from the best model were defined as the DLB-pattern. The horizontal dotted line denotes 5% explained in the principal component analyses. The receiver operating characteristic curve for differentiation of DLB patients from healthy controls with DLB-pattern is presented as an  inset figure

This study included a total of 141 participants including 44 healthy controls, 50 iRBD patients, 22 DLB patients and 25 AD (Fig. 1a). The baseline clinical features of the healthy controls, iRBD patients and DLB patients are described in Table 1. The DLB group was older than the healthy control and the iRBD groups (Kruskal–Wallis test, *P* = 6.37 × 10^–5^; Table 1). MMSE scores were significantly lower in the DLB group than in the healthy control and the iRBD groups (Kruskal–Wallis test, *P* = 2.62 × 10^–9^, Table 1). Total MDS-UPDRS part I, II and III scores were higher in DLB patients compared to iRBD patients (rank-sum test, *P* < 0.01). The DLB group showed significantly worse performance in the Boston naming test, Rey figure copy test, verbal learning test and TMT-B compared to the healthy control and iRBD groups (Table 1). The clinical features of the AD group (*n* = 25; age: 71.4 ± 4.9 years; disease duration: 1.8 ± 1.3 years; male/female: 11/14; MMSE: 22.8 ± 4.4; CDR: 0.6 ± 0.2) were collected.

### Comparison of cortical thickness among DLB, iRBD and healthy control

Direct comparison of cortical thickness in DLB patients and age-matched controls showed significant cortical thinning in prefrontal, medial frontal, paracentral lobule, cingulate, precuneus, middle temporal, temporopolar, insular, fusiform, and occipital cortices (corrected *P* < 0.01; Fig. 1b). Compared with the iRBD group, DLB group also showed diffuse cortical thinning in prefrontal, medial frontal, middle temporal, temporopolar, insular and occipital cortices (corrected *P* < 0.01; Additional file 1: Fig. S2). However, there was no definite difference in cortical thickness between iRBD and healthy controls after multiple corrections.

### Characterization of the DLB-related covariance pattern of cortical thickness

**Fig. 2:**
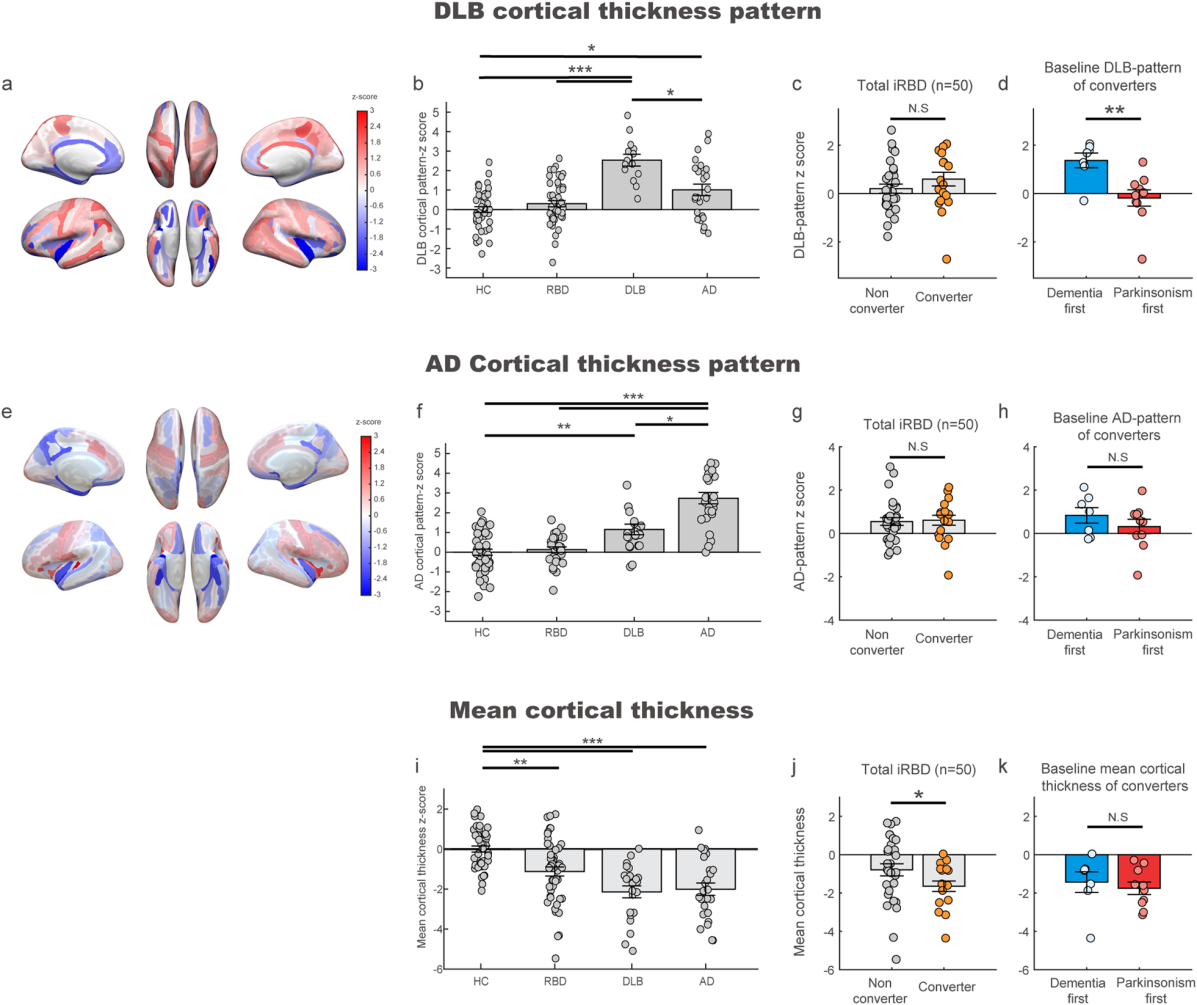
Topography of cortical thickness signature and its expression in different groups including converters/non-converters in iRBD. **a** Spatial map representing the DLB-pattern derived from DLB patients and healthy controls. Red and blue colors represent positive and negative contributions to the DLB-pattern, respectively. **b** Group comparison of DLB-pattern scores across different groups. The DLB-pattern scores were normalized with the mean and standard deviation of healthy controls. The thick horizontal lines and error bars represent the mean and standard error mean (sem), respectively. The *P* values were calculated with Kruskal–Wallis test with Dunn’s *post-hoc* test (*P < 0.05, *** *P* < 0.001). **c** Distribution of the baseline DLB-pattern scores of future converters (orange) and non-converters (gray) in total iRBD patients. Rank-sum test, N.S: not significant. **d** Distribution of the baseline DLB-pattern scores in future dementia-first converters (blue) and parkinsonism-first converters (red) in the iRBD cohort. (rank-sum test, **P < 0.01). **e** Spatial map representing the AD-related cortical thickness covariance pattern (AD-pattern) derived from AD patients and healthy controls. Red and blue colors represent positive and negative contributions to the AD-pattern pattern, respectively. **f** Group comparison of the AD-pattern scores across different groups. The error bar represents the mean and standard error mean (sem), respectively. The *P* values were calculated with Kruskal–Wallis test with Dunn’s *post-hoc* test. (*P < 0.05, ** *P* < 0.01, *** *P* < 0.001). **g** Distribution of the baseline AD-pattern scores of future converters (orange) and non-converters (gray) in total iRBD patients. (rank-sum test, N.S.: not significant). **h** Distribution of the baseline AD-pattern scores in future dementia-first converters (blue) and parkinsonism-first converters (red) in the iRBD cohort (rank-sum test, N.S.: not significant). **i** Group comparison of mean cortical thickness across different groups. The error bar represents the mean and standard error mean (sem), respectively. The *P* values were calculated with Kruskal–Wallis test with Dunn’s *post-hoc* test. (**P < 0.01, *** *P* < 0.001). **j** Distribution of baseline mean cortical thickness scores of future converters (orange) and non-converters (gray) in total iRBD patients. Rank-sum test, *P < 0.05. **k** Distribution of baseline mean cortical thickness scores in future dementia-first converters (blue) and parkinsonism-first converters (red) in the iRBD cohort. Rank-sum test, N.S.: not significant

A covariance matrix of cortical thickness for DLB patients and age-matched control samples was calculated, followed by PCA analysis (Fig. 1c). From the first 5 PCs that accounted for 64.1% of the variance in the spatial map of cortical thickness, the combination of PCs 1, 2, 3 and 5 resulted in the best performance in differentiating DLB patients from healthy controls (area under the curve [AUC] = 0.962; Fig. 1c). The spatial weights from the best model were defined as the DLB-related covariance pattern of cortical thickness (DLB-pattern). The DLB-pattern was negatively contributed by the medial temporal, anterior temporal, orbitofrontal, and insula cortices and positively contributed by the precentral and inferior parietal cortices (absolute z-scores > 1.5, Fig. 2a). We confirmed that the ROIs representing the spatial cortical pattern of DLB were stable based on bootstrap resampling (*P* < 0.05; Additional file 1: Fig. S1). The DLB-pattern scores were significantly lower in healthy control, iRBD and AD groups compared with the DLB group (*post-hoc P* < 0.05; Fig. 2b). Among the 50 iRBD patients, 19 phenoconverted during the follow-up (10 to PD, 7 to DLB and 2 to MSA). The average follow-up duration (from baseline evaluation until the last contact) was 4.43 ± 2.59 years and the total person-years of the iRBD group was 217.2. The annual conversion rate was 8.25% estimated by Kaplan–Meier analyses (Additional file 1: Fig. S3). The distribution of baseline DLB-pattern scores was not different between future converters and non-converters (Fig. 2c). However, the baseline DLB-pattern scores were significantly higher in dementia-first than in parkinsonism-first converters (rank-sum test, *P* = 0.0068; Fig. 2d). The cut-off value of 1 standard deviation from healthy controls differentiated dementia-first and parkinsonism-first converters in iRBD with the best performance (AUC = 0.938).

### Characterization of AD-related cortical thickness and correlation with the DLB-pattern

The AD-pattern was based on the best performance in differentiating AD patients from age-matched controls (AUC = 0.872). The AD-pattern represented an overall negative association with the temporal, parietal, insular and orbitofrontal cortices (Fig. 2e). Expression of the AD-pattern was significantly higher in the AD group compared to all other groups (*post-hoc* *P* < 0.05, Fig. 2f). The AD-pattern scores did not differ between converters and non-converters in the iRBD cohort or between dementia-first and parkinsonism-first phenoconverters of iRBD. (Fig. 2g, h). Despite the differences in pattern expression in individual iRBD subjects, the DLB-pattern and AD-pattern scores correlated with each other in study participants (Pearson’s correlation, *R* = 0.49, *P* = 2.5 × 10^–14^; Additional file 1: Fig. S4).

### Mean value of whole-brain cortical thickness

We estimated the mean value of the whole-brain cortical thickness in every individual and analyzed the group differences. The mean cortical thickness was significantly lower in the DLB and AD patients compared to the healthy controls (*post-hoc P* < 0.001) and was similar between the DLB and AD groups (*post-hoc P* = 0.99; Fig. 2i). The mean value of whole-brain cortical thickness was lower in future converters than in non-converters (rank-sum test, *P* = 0.045; Fig. 2j) among the iRBD population. The mean cortical thickness z-score of 0 as a cut-off fairly differentiated converters from non-converters (AUC = 0.702). However, the values of mean cortical thickness were not different between the dementia-first and parkinsonism-first phenoconverters (rank-sum test, *P* = 0.42; Fig. 2k). The mean cortical thickness was negatively correlated with age (*R* = − 0.30, *P* = 0.044).

### Clinical relevance of the DLB-pattern, AD-pattern and mean cortical thickness

In the DLB patients and healthy controls, DLB-pattern scores showed negative correlation with TMT-A (*R* = − 0.55, *P* = 0.024), TMT-B (*R* = − 0.56, *P* = 0.036), and RCFT (*R* = − 0.54, *P* = 0.0047) after adjusting for age, sex, and education years (Table 2). In contrast, the MDS-UPDRS I, II, and III scores and cognition subscore (MDS-UPDRS I.1) did not correlate with the DLB-pattern scores (Table 2). The mean cortical thickness significantly correlated with the MDS-UPDRS cognition subscore (*R* = − 0.64, *P* = 4.18 × 10^–4^), MDS-UPDRS I (*R* = − 0.45, *P* = 0.017), II (*R* = − 0.52, *P* = 0.0045), III (*R* = − 0.58, *P* = 0.0029), BNT (*R* = 0.48, *P* = 0.013) and SVLT recognition scores (*R* = 0.50, *P* = 0.0078) with age, sex and education years as cofactors. In contrast, AD-pattern scores did not correlate with any clinical or cognition score in the DLB patients.

**Table 2:** Correlation of cortical thickness signature with clinical parameters in derivation samples of age-matched DLB and healthy controls

	DLB-pattern		AD-pattern		Mean cortical thickness	
	Correlation coefficient	*P* value	Correlation coefficient	*P* value	Correlation coefficient	*P* value
MMSE	− 0.31	0.035	− 0.088	0.55	0.37	0.011
Cognition (MDS-UPDRS part I)	0.051	0.79	0.17	0.41	− **0.64**	**4.18 × 10**^**–4**^
MDS-UPDRS part I	− 0.042	0.92	0.12	0.52	− **0.45**	**0.017**
MDS-UPDRS part II	0.041	0.90	− 0.16	0.42	− **0.52**	**0.0045**
MDS-UPDRS part III	0.051	0.87	0.24	0.26	− **0.58**	**0.0029**
Digit span test	− 0.11	0.58	0.22	0.28	0.053	0.80
TMT-A	− **0.55**	**0.024**	− 0.081	0.79	0.058	0.84
CWST	− 0.24	0.32	− 0.26	0.35	− 0.11	0.69
BNT	− 0.35	0.062	− 0.095	0.65	**0.48**	**0.013**
RCFT	− **0.54**	**0.0047**	− 0.19	0.38	0.33	0.12
SVLTi	− 0.11	0.56	− 0.065	0.75	**0.37**	**0.060**
SVLTd	− 0.25	0.18	− 0.089	0.66	0.32	0.10
SVLTr	− 0.16	0.41	0.14	0.49	**0.50**	**0.0078**
COWAT	0.083	0.73	0.38	0.16	− 0.028	0.92
TMT-B	− **0.56**	**0.036**	− 0.29	0.33	0.24	0.41

Age and sex were included as cofactors for correlation analysis between cortical thickness pattern score and clinical variables MDS-UPDRS part I (except for cognition subscore), II and III. Age, sex and education year were included as cofactors in the correlation analysis between cortical thickness patterns and cognitive profiles (Cognition subscore from MDS-UPDRS part I and scores from neuropsychological tests)

The significant values (*P* < 0.05) are shown in bold

*BNT* Boston Naming Test, *COWAT* Controlled Oral Word Association Test, *CWST* Korean Color Word Stroop Test, *DLB* Dementia with Lewy bodies, *MDS-UPDRS* Movement Disorders Society-Unified Parkinson Disease Rating Scale, *RCFT* Rey Complex Figure Test copy, *SVLT* Seoul Verbal Learning Test, *TMT* Trail Making Test

In the iRBD group, we observed a negative correlation between the DLB-pattern scores and digit span test scores (*R* = − 0.32, *P* = 0.033; Table 3). In contrast, the AD-pattern scores and the mean cortical thickness did not correlate with any baseline clinical or neuropsychological profile in iRBD patients (Table 3). There was a significant negative correlation between the baseline DLB-pattern and 4-year longitudinal changes of RCFT (*R* = − 0.22, *P* = 0.035) and SVLT immediate recall scores (*R* = − 0.46, *P* = 0.018) controlled for age, sex and education years (Table 3). The AD-pattern scores significantly correlated with the 4-year longitudinal changes of immediate (*R* = − 0.46, *P* = 0.048) and delayed recall scores (*R* = − 0.60, *P* = 0.007) on the SVLT test in the iRBD patients with age, sex and education years as cofactors.

**Table 3:** Correlations of cortical thickness signature scores at baseline with clinical parameters at baseline and at 4-year follow-up in iRBD

	DLB-pattern				AD-pattern				Mean cortical thickness			
	Baseline		4-year progression		Baseline		4-year progression		Baseline		4-year progression	
	Correlation coefficient	*P*	Correlation coefficient	*P*	Correlation coefficient	*P*	Correlation coefficient	*P*	Correlation coefficient	*P*	Correlation coefficient	*P*
B-SIT	− 0.14	0.40	N.A	N.A	− 0.19	0.24	N.A	N.A	0.039	0.21	N.A	N.A
Cognition*	− 0.017	0.91	0.026	0.92	0.23	0.13	0.19	0.44	− 0.086	0.57	0.054	0.83
MDS-UPDRS part I	0.14	0.36	− 0.28	0.28	0.27	0.069	− 0.19	0.44	− 0.11	0.46	− 0.31	0.20
MDS-UPDRS part II	0.13	0.39	− 0.11	0.67	0.27	0.065	0.15	0.55	− 0.11	0.47	− 0.19	0.45
MDS-UPDRS part III	0.08	0.56	− 0.044	0.25	0.16	0.29	0.33	0.17	− 0.18	0.23	− 0.26	0.28
Digit span test	− **0.32**	**0.033**	0.29	0.61	− 0.036	0.82	0.064	0.81	− 0.039	0.80	0.10	0.70
TMT-A	− 0.21	0.21	0.13	0.067	− 0.12	0.47	0.057	0.83	− 0.084	0.61	0.33	0.21
CWST	0.31	0.075	− 0.43	0.31	− 0.11	0.54	− 0.41	0.12	0.11	0.54	− 0.13	0.62
BNT	0.042	0.79	− 0.23	0.37	− 0.019	0.90	− 0.22	0.37	− 0.26	0.082	− 0.088	0.72
RCFT	− 0.067	0.68	− **0.22**	**0.035**	0.17	0.26	− 0.34	0.17	− 0.22	0.16	− 0.0062	0.98
SVLTi	0.073	0.65	− **0.46**	**0.018**	0.25	0.097	− **0.46**	**0.048**	0.080	0.60	− 0.085	0.73
SVLTd	0.061	0.70	− 0.51	0.068	0.12	0.42	− **0.60**	**0.007**	0.16	0.29	− 0.44	0.057
SVLTr	− 0.073	0.64	− 0.41	0.54	0.18	0.25	− 0.24	0.33	− 0.17	0.27	0.23	0.35
COWAT	0.067	0.68	− 0.16	0.54	0.0076	0.96	− 0.19	0.49	0.022	0.89	0.11	0.69
TMT-B	0.25	0.13	− 0.002	0.99	0.10	0.58	0.32	0.24	− 0.052	0.78	0.29	0.29

Age and sex were included as cofactors for correlation analysis between cortical thickness signature scores (DLB-pattern, AD-pattern and mean cortical thickness) and clinical variables (SIT, MDS-UPDRS part I, II and III). Age, sex and education year were included as cofactors in the correlation analysis between cortical signature scores with cognitive profiles (Cognition* from MDS-UPDRS part I subscore and scores from neuropsychological tests)

The significant values (*P* < 0.05) are shown in bold

*B-SIT* Brief smell identification test, *BNT* Boston Naming Test, *COWAT* Controlled Oral Word Association Test, *CWST* Korean Color Word Stroop Test, *SIT* Smell identification test, *MDS-UPDRS* Movement Disorders Society-Unified Parkinson Disease Rating Scale, *TMT* Trail Making Test, *RCFT* Rey Complex Figure Test copy, *SVLTi* SVLTd, SVLTr: immediate recall, delayed recall and recognition in Seoul Verbal Learning Test, respectively. *N.A*. Not Applicable

### Longitudinal analyses of the cortical thickness signature in iRBD

**Fig. 3:**
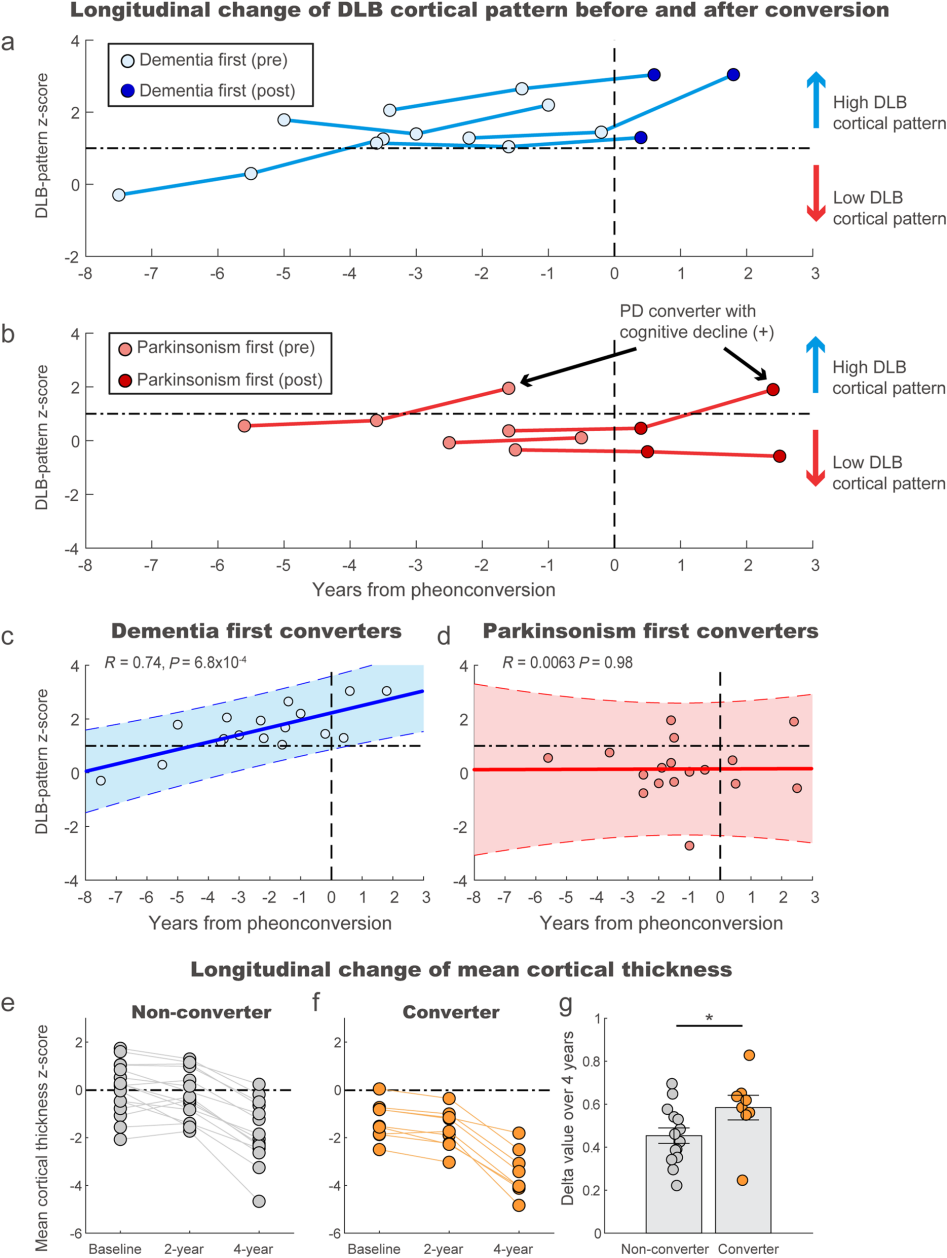
Longitudinal analyses of cortical thickness signature over 4 years of follow-up in iRBD. **a** Longitudinal change of individual DLB-pattern scores in dementia-first converters. Patients with the pre-conversion state are marked with a light blue color and patients after conversion are marked with dark blue. The time of phenoconversion was marked with a vertical dotted line. The best cut-off discriminating future dementia-first and parkinsonism-first converters is  marked as a horizontal dotted line (1 standard deviation from healthy control, AUC = 0.938). **b** Longitudinal change of individual DLB-pattern scores in parkinsonism-first converters as the same format in **a**. **c, d** Progression of DLB-pattern scores from prodromal stage to the converted stage in dementia-first converters (**c**) and parkinsonism-first converters (**d**). The shading represents 95% confidence. The vertical dotted line represents the timing of conversion. The horizontal dotted line is from the cut-off value in **a**. **e, f** Longitudinal change of mean cortical thickness from the baseline to 2-year and 4-year follow-ups in non-converters (**e**) and converters (**f**). **g** Comparison of the delta value (change of mean cortical thickness over 4 years) between non-converters and converters in iRBD patients (rank-sum test, *P < 0.05)

In the subpopulation of iRBD patients (*n* = 31 including 5 DLB- and 4 PD-converters), we repeated MRI scans and clinical evaluations at 2 and 4 years from the baseline. All dementia-first converters showed high DLB-pattern scores above the cut-off (≥ 1 z-score) at least within 4 years of phenoconversion (Fig. 3a). The parkinsonism-first converters showed low DLB-pattern scores (< 1 z-score) at baseline and follow-ups (Fig. 3b). There were two parkinsonism-first converters who later showed elevations of the DLB-pattern to above the cut-off during the additional follow-up longer than 4 years. These patients developed cognitive decline at later follow-ups, as evaluated with a comprehensive neuropsychological battery according to the cohort protocol described in the method. The DLB-pattern scores significantly increased during longitudinal follow-up (*R* = 0.74; *P* = 6.8 × 10^–4^) in dementia-first converters and exceeded the predictive cut-off (z-score > 1) at 4.1 years prior to phenoconversion (Fig. 3c). In contrast, the DLB-pattern scores remained below the cut-off in parkinsonism-first converters throughout the follow-up (*R* = 0.0063; *P* = 0.98, Fig. 3d). In non-converters, our analysis showed that the DLB-cortical thickness pattern remained stable over time in majority of the cases. However, individual patterns were heterogenous and some non-converter iRBD patients exhibited a pattern of increase over the 4-year period (Additional file 1: Fig. S5). In the longitudinal analyses of mean cortical thickness in the iRBD patients, both the non-converters and converters showed ongoing atrophy over 4 years (Fig. 3e, f). However, the delta value (i.e., change in the mean cortical thickness z-scores over 4 years) was significantly greater for the converters than for the non-converters (rank-sum test; *P* = 0.044, Fig. 3g).

### Prediction of phenoconversion with the cortical thickness signature in iRBD patients

**Fig. 4:**
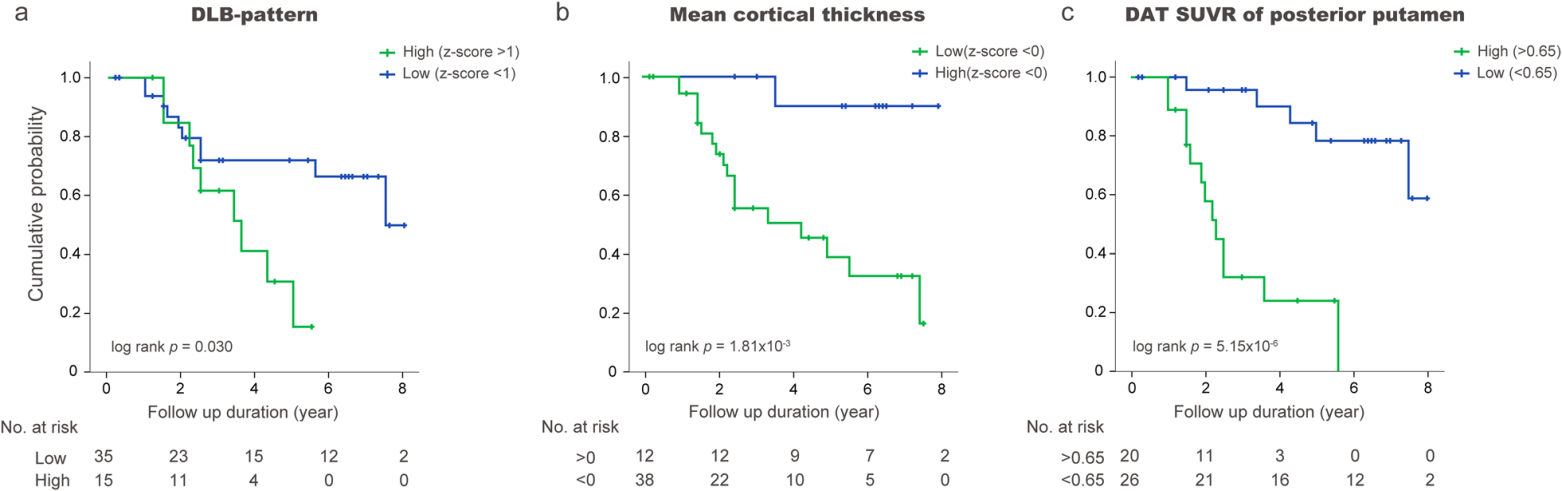
Prediction of disease conversion with baseline DLB-pattern, mean cortical thickness and DAT availability. **a** Kaplan–Meier plot for overall disease conversion in iRBD patients with DLB-pattern [green: high z-score(> 1); blue: low z-score(< 1)]. **b** Kaplan–Meier plot for overall disease conversion in iRBD with the mean cortical thickness [green: low z-score(< 0); blue: high z-score(> 0)]. **c** Kaplan–Meier plot for overall disease conversion in iRBD with decreased DAT-SUVR (age-normative value of posterior putamen < 0.65). SUVR: Standardized uptake value ratio, DLB: Dementia with Lewy bodies, DAT: Dopamine transporter

The DLB-pattern did not significantly predict overall disease conversion (Table 4; Fig. 4a). In contrast, mean cortical thickness showed significant predictive performance (HR [95% confidence interval] = 9.33 [1.16, 74.12]) which was comparable to reduced DAT availability (DAT standardized uptake value ratio [SUVR] < 0.65 of age-normative value in the posterior putamen, HR = 8.65 [2.54, 29.41]) (Table 4; Fig. 4b, c). Among clinical biomarkers, hyposmia showed a significant prediction of overall phenoconversion (HR = 4.38 [1.47, 13.08]) in our iRBD cohort (Table 4) [1].

**Table 4:** Performance of cortical thickness signature scores with the hazard ratio for total conversion and sensitivity/specificity for differentiating dementia-first vs. motor-first conversion in iRBD

Total disease conversion in iRBD	Hazard ratio [95% Confidence interval]
MRI (DLB-pattern)	2.27 [0.70, 7.61]
MRI(Mean cortical thickness)	9.33 [1.16, 74.12]
^18^F-FP-CIT PET (posterior putamen SUVR < 0.65)	8.65 [2.54, 29.41]
Hyposmia (B-SIT)	4.38 [1.47, 13.08]
Objective motor examination*	2.89 [0.91, 9.18]

Dementia-first (*n* = 7) vs Parkinsonism-first (*n* = 10) among the converters	Overall discrimination		
	Sensitivity (%)	Specificity (%)	Diagnostic accuracy (%)
MRI (DLB-pattern)	85.7	90	88.2
MRI (Mean cortical thickness)	85.7	0	35.3
^18^F-FP-CIT PET (posterior putamen SUVR < 0.65)	57.1	10	29.4
Hyposmia (B-SIT)	66.7	33.3	31.3
Objective motor examination*	85.7	30.0	58.2

*Objective motor examination was defined by UPDRS part III score > 3 excluding action tremor[1]

*AD* Alzheimer’s dementia, *B-SIT* Brief smell identification test, *DLB* Dementia with Lewy bodies, *iRBD* Idiopathic rapid-eye-movement sleep behavior disorder, *SUVR* Standardized uptake ratio

Sensitivity, specificity and diagnostic accuracy were calculated by detection of dementia-first converters among total converters as follows

a = number of dementia-first converters with positive biomarker

b = number of total dementia-converters

c = number of motor-first converters with negative biomarker

d = number of total motor-first converters

(a/b for sensitivity, c/d for specificity and (a + c)/(a + b + c + d) for diagnostic accuracy)

The baseline DLB-pattern significantly differentiated dementia-first from parkinsonism-first converters with excellent sensitivity (85.7%), specificity (90%) and diagnostic accuracy (88.2%), while the DAT-SUVR or mean cortical thickness did not (Table 4). The DLB-pattern values obtained within 4 years of phenoconversion showed even higher sensitivity (100%), specificity (90%), and diagnostic accuracy (94.1%) in discriminating dementia-first from parkinsonism-first converters in iRBD. None of the clinical biomarkers were able to discriminate dementia-first from parkinsonism-first converters in the iRBD cohort (Table 4).

## Discussion

### Topography of the DLB-pattern and AD-pattern

The DLB-pattern was derived from the model that successfully differentiated DLB from healthy controls (AUC = 0.962). There have been attempts to differentiate DLB from healthy controls based on structural imaging (MRI) with single or multiple ROIs [25–27]. The performance of DLB-pattern in this study was comparable to the previous study combining atrophy in multiple ROIs and small vessel disease burden [26]. Topographically, the bilateral inferior insula thickness was negatively associated with the DLB-pattern (z-score = − 1.90 and − 2.35 in the left and right inferior insula, respectively), which survived the boot-strap validation. Atrophy in the insular cortex in DLB has been consistently reported by independent study groups [28–30], which is also evident in prodromal DLB patients [8, 31]. Of note, the insula is the anatomical substrate responsible for cognitive fluctuation, hallucination, and autonomic dysfunction, which are the core features of DLB [32–34]. Moreover, in this study, cingulate, orbitofrontal, and inferior temporal cortices negatively contributed to the DLB-pattern, which was consistent with previous cross-sectional studies [8].

The AD-pattern was similar to the DLB-pattern in terms of the negative associations with the insula, medial temporal and orbitofrontal cortices. Considering the overlapping pathological findings between DLB and AD [35], the neurodegeneration pattern derived from DLB patients might share some characteristics with AD. This was supported by the significant correlation between the AD-pattern z-score and DLB-pattern z-score among participants (Additional file 1: Fig. S4). Furthermore, DLB-related cortical thickness scores calculated in the AD group were increased compared to the healthy control although lower than the DLB group. However, the negative associations with the superior parietal, precuneus and middle/inferior temporal cortices were a distinct feature of the AD-pattern, consistent with the previous literature reporting a cortical thinning signature of AD [36]. Further studies applying the AD- and DLB-patterns to various stages of AD or DLB are needed to elucidate how the cortical thickness pattern may indicate disease progression from the prodromal stage towards AD or DLB.

### Clinical and neuropsychological correlates of cortical thickness signatures: longitudinal analyses

The DLB-pattern showed a significant negative correlation with attentional and frontal executive function (TMT-A and TMT-B) and visuospatial function (RCFT), which reflect the neuropsychological characteristics of DLB (Table 2) [37]. We observed that the neuropsychological correlates of the DLB-pattern in the iRBD group were worse performance in RCFT and SVLT during the follow-up (Table 3). A distinguishing feature of DLB from PD-MCI is memory impairment [38, 39]. Therefore, the correlation between the DLB-pattern scores and the progression of impaired verbal memory in iRBD was consistent with the observation that the DLB-pattern scores were not elevated in the majority of parkinsonism-first converters. Furthermore, although the DLB-pattern could not differentiate iRBD patients with MCI (iRBD-MCI) from iRBD without MCI, it significantly differentiated iRBD-MCI who later converted to DLB from iRBD-MCI who later converted to PD. The longitudinal evolution in DLB-pattern expression in the dementia-first converters was noticeable based on its absence in parkinsonism-first converters even during the follow-ups. Our results suggest that the DLB-pattern is the specific cortical pattern reflecting prodromal neurodegeneration related to DLB in iRBD. Importantly, parkinsonism-first converters who later developed cognitive impairment showed elevated DLB-pattern scores in the longer follow-up (Fig. 3b). Therefore, the DLB-pattern may be associated with cognitive impairment in the spectrum of DLB and Parkinson disease dementia (PDD). The presence of RBD has been found to be associated with a higher degree of cognitive dysfunction and cortical degeneration in various populations, including aged healthy individuals, iRBD patients, and PD [4, 40, 41]. Future research is needed to investigate the potential association of the DLB-pattern in PDD patients, including cases evolving from the iRBD population. The DLB-cortical thickness pattern remained stable over time in a majority of the non-converter iRBD patients. However, the individual patterns were heterogeneous, and some showed an increasing pattern over 4 years, suggesting subclinical neurodegeneration towards dementia-first phenoconversion in these patients. Extended follow-ups may reveal more converters, especially those with dementia-first conversion.

Consistent with a previous longitudinal study in a large cohort [42], the mean cortical thickness showed a negative correlation with age in the healthy controls. In contrast, the DLB-pattern did not correlate with age in the healthy controls. The mean cortical thickness scores significantly correlated with overall motor and non-motor features (UPDRS part I, II and III scores) in DLB. Thus, the mean cortical thickness may reflect the overall disease severity in DLB, which corresponds with previous studies showing the correlation of cortical thickness with disease stages in DLB [43].

### The cortical thickness signature and phenoconversion in iRBD

In this study, the low value of mean cortical thickness at baseline was found to significantly predict overall phenoconversion. This result is in agreement with a previous study by Pereira et al., showing that the mean cortical thickness in ROIs that differed between converters and non-converters, significantly predicted overall phenoconversion in iRBD [13]. The baseline DLB-pattern differentiated dementia-first converters from parkinsonism-first converters with sensitivity and specificity of 85.7% and 90%, respectively. Thus, the complementary role of cortical thickness signature of mean cortical thickness and the DLB-pattern acquired from a single modality of MRI may stratify subtype-specific phenoconversion in iRBD.

In an effort to stratify LBD subtypes in iRBD patients, Rahayel et al. derived a clinical-imaging signature from iRBD patients that could predict dementia-first phenoconversion [44]. The clinical-imaging signature was derived using the partial least squares method, which involved analyzing the correlation matrix between the clinical matrix and the whole-brain deformation-based morphometry matrix. In contrast, we used the subprofile model of principal components analysis to identify a cortical thickness pattern, using only cortical thickness data estimated in 148 ROIs. Another important difference between our approach and that of Rahayel et al. is that they derived the imaging signature from the iRBD patients, while we derived the cortical thickness pattern from patients with DLB. Despite this methodological difference, both studies showed that a whole-brain structural signature, whether derived from the iRBD or DLB group, may reflect prodromal neurodegeneration leading to the onset of dementia in iRBD. Another study by Arnaldi et al. showed that asymmetry in caudate binding in DAT-SPECT combined with MMSE scores might be able to differentiate dementia-first from parkinsonism-first converters although the predictability did not reach statistical significance [45]. However, these analytic methods including ours were data-driven approaches; therefore, replications in independent iRBD cohorts are needed. In addition to imaging markers, serum metabolic biomarkers regarding glycosylation and lipid profiles also showed excellent sensitivity and specificity [46], but accessibility to these tests is limited and thus difficult to generalize across the iRBD population. One study suggested that the pareidolic illusion may potentially indicate high-risk DLB phenoconversion in iRBD [47]. However, more studies with longitudinal evaluations are needed to address the use of pareidolia for phenoconversion prediction in iRBD.

In our study, none of the clinical biomarkers were able to discriminate dementia-first from parkinsonism-first conversion (Table 4). These findings suggest the strength of imaging biomarkers in predicting subtypes of prodromal LBD in iRBD. Furthermore, our approach has the advantage that the matrix of the DLB-pattern obtained in this study can be easily applied to any MRI data obtained from the iRBD population to derive the expression z-score on an individual basis. As MRI is a widely available imaging modality, the cortical thickness signature can be easily applied to patients across clinics around the world.

The current study has some limitations. First, the MRI-driven DLB-related cortical thickness pattern needs to be reproduced in large multicenter cohorts. Second, large-sample studies with longer follow-ups would be valuable to evaluate the relationship between the cortical signature and various prodromal markers comprehensively. Nevertheless, the sample size in this study was acceptable for analyzing the predictive performance of our imaging markers because of the relatively high HRs of these markers [48]. Third, the rare phenoconversion to MSA was not analyzable with our data. The identification of MSA-predicting biomarkers in iRBD may require a combination of different arrays of biomarkers which should be elucidated with larger multicenter cohorts in the future.

## Conclusions

The MRI-driven DLB-pattern reflects prodromal clinical features and longitudinal evolution of Lewy body dementia in individuals with iRBD. The MRI-based index consisting of the mean cortical thickness values and the DLB-pattern expression score would help predict and monitor DLB-type phenoconversion in individual patients with iRBD.

## Supplementary Information

**Additional file 1: Fig. S1.** Stable regions of interestin DLB-related cortical thickness pattern. **Fig. S2.** Direct comparison of the cortical thickness for iRBD versus DLB. **Fig. S3.** Disease-free survival in iRBD patients with Kaplan-Meier analysis. **Fig. S4.** Correlation between the DLB-pattern and the AD-pattern. **Fig. S5.** Longitudinal change of DLB-pattern in the non-converter iRBD group. **Table S1.** List of ROIs for cortical thickness analysis.

## Data Availability

The datasets and codes generated during the current study are available from the corresponding author on reasonable request including reproducibility of research or external validation. Restrictions may be applied to sensitive data for privacy preservation.

## Abbreviations

iRBD: Isolated rapid-eye-movement sleep behavior disorder
PD: Parkinson’s disease
MSA: Multiple system atrophy
DLB: Dementia with Lewy bodies
SUVR: Standardized uptake value ratio
TMT: Trail making test
AD: Alzheimer’s dementia
CDR: Clinical dementia rating
MDS-UPDRS: Movement disorders society revised-unified PD rating scale
MMSE: Mini-mental status exam
SVLT: Seoul verbal learning test
RCFT: Rey complex figure test
HR: Hazard ratio
MCI: Mild cognitive impairment
DAT: Dopamine transporter
