# Supplementary material for: Longitudinal evolution of cortical thickness signature reflecting Lewy body dementia in isolated REM sleep behavior disorder: a prospective cohort study
Source: Transl Neurodegener. 2023 May 22;12:27. doi: 10.1186/s40035-023-00356-y (PMC10201719; doi:10.1186/s40035-023-00356-y)
Supplement: Supplementary file 1 — Additional file 1: Fig. S1. Stable regions of interestin DLB-related cortical thickness pattern. Fig. S2. Direct comparison of the cortical thickness for iRBD versus DLB. Fig. S3. Disease-free survival in iRBD patients with Kaplan-Meier analysis. Fig. S4. Correlation between the DLB-pattern and the AD-pattern. Fig. S5. Longitudinal change of DLB-pattern in the non-converter iRBD group. Table S1. List of ROIs for cortical thickness analysis. [file 40035_2023_356_MOESM1_ESM.docx]

**Additional file 1**

**
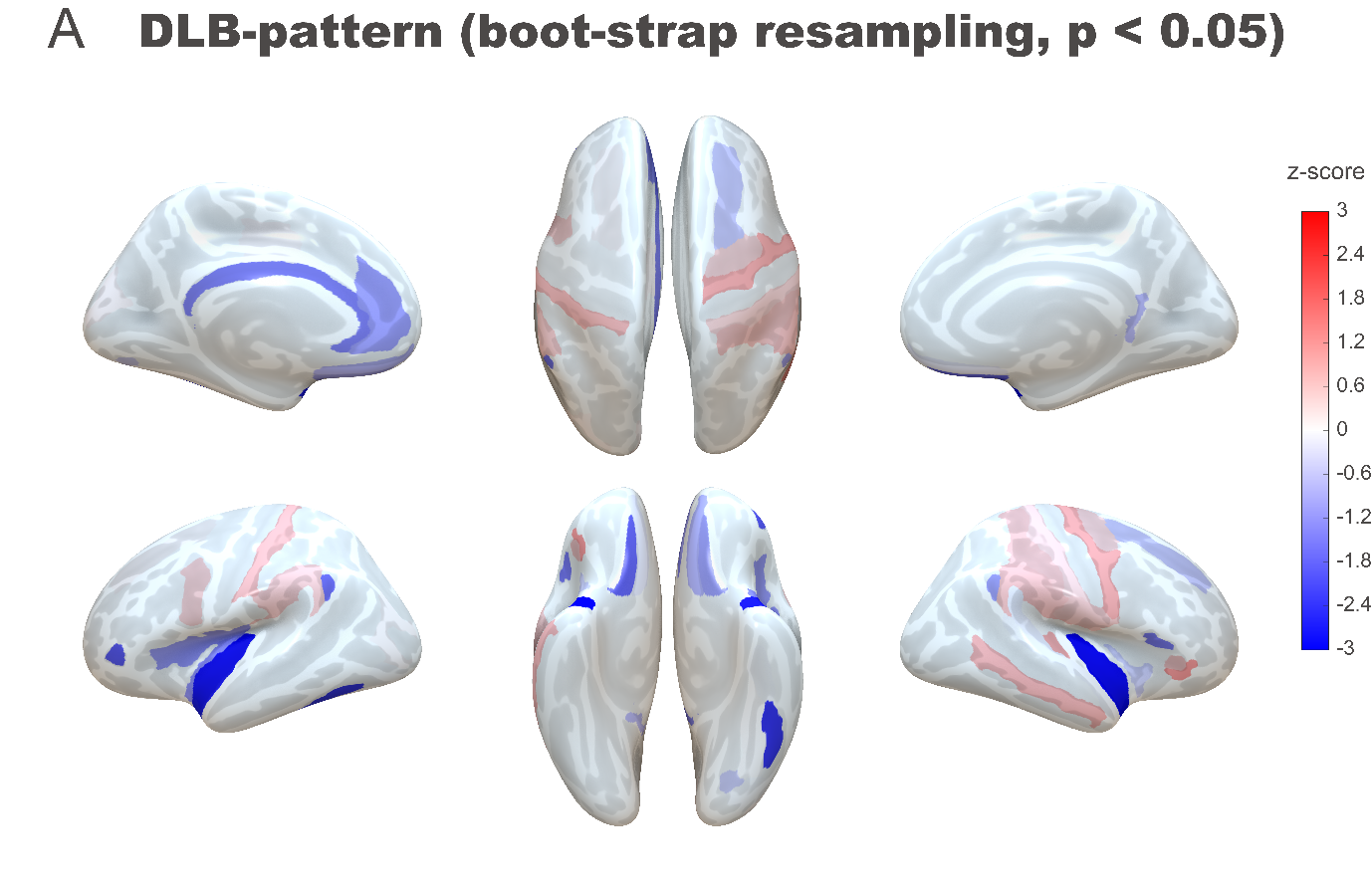
**

**Figure S1. Stable regions of interest (ROIs) in DLB-related cortical thickness pattern (DLB-pattern).** Spatial map representing the DAT-pattern with stable ROIs (boot-strap resampling x 10000 times, p < 0.05) Red and blue colors represent positive and negative contributions to the DAT-pattern, respectively.


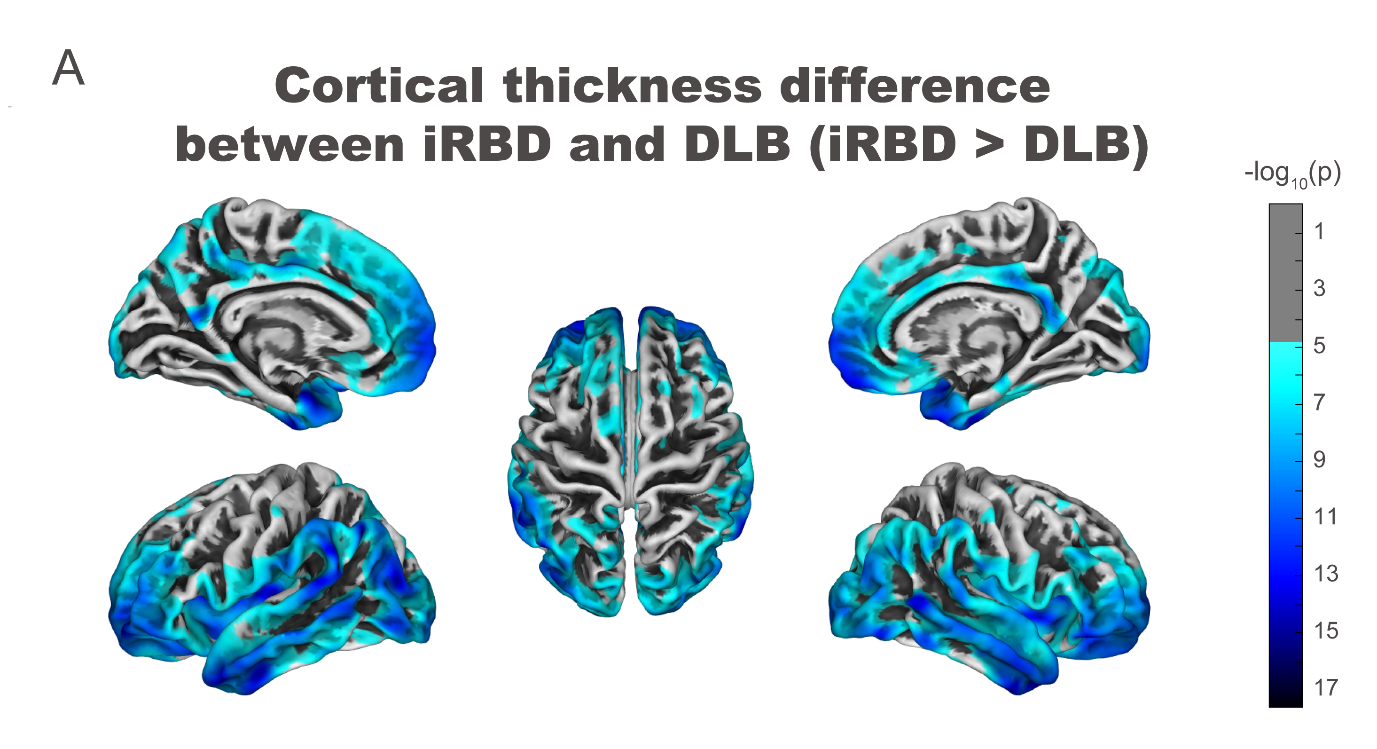


**Figure S2. Direct comparison of the cortical thickness for iRBD versus DLB.** Cortical thinning in DLB patients compared with iRBD showed significant thinning in bilateral temporal, frontal, parietal, and occipital cortices (corrected *P<*0.01).

**
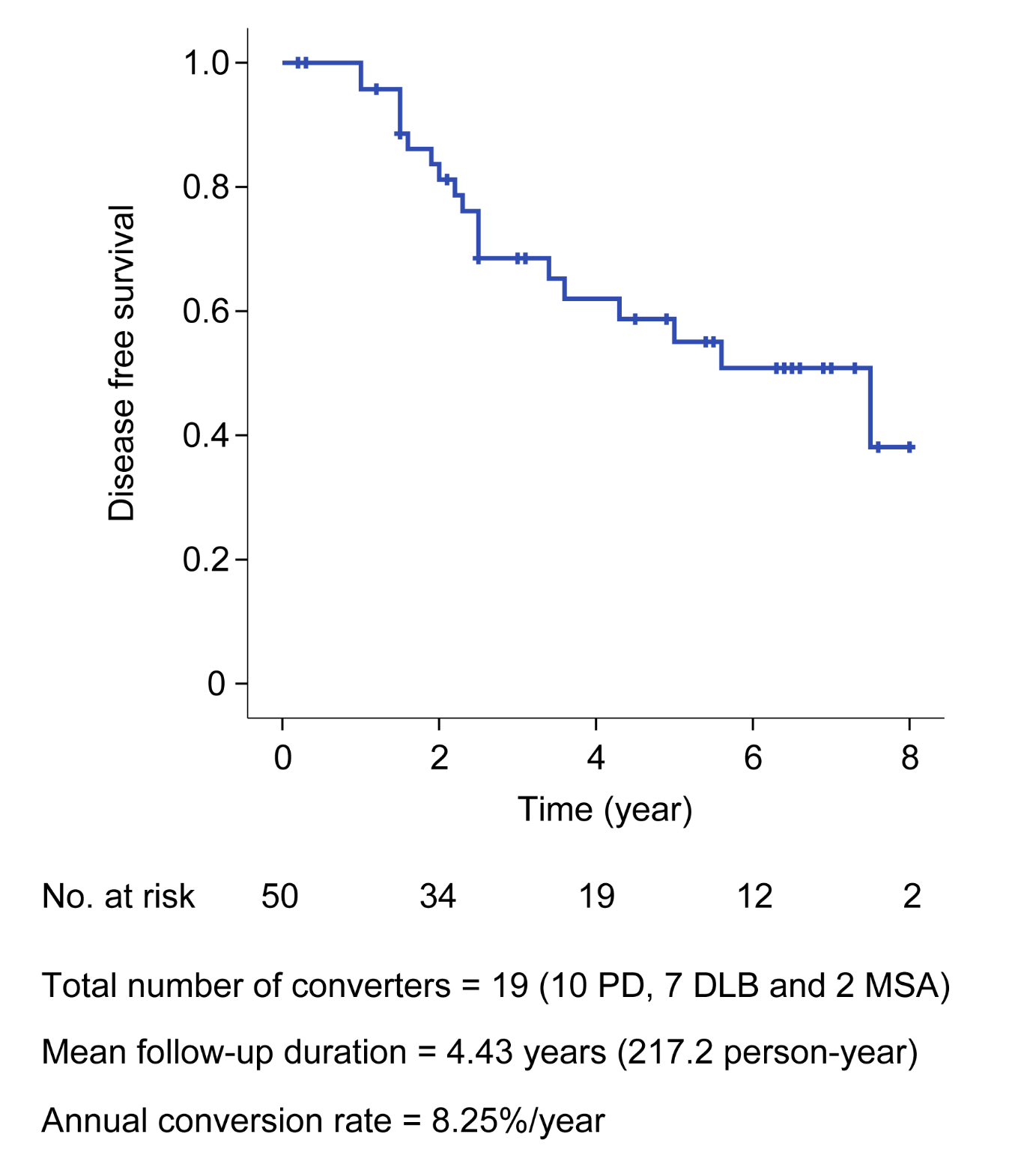
**

**Figure S3. Disease-free survival in iRBD patients with Kaplan-Meier analysis.** Abbreviations. iRBD=idiopathic rapid eye movement sleep behavior disorder; PD=Parkinson’s disease; DLB=dementia with Lewy bodies; MSA=multiple system atrophy

**
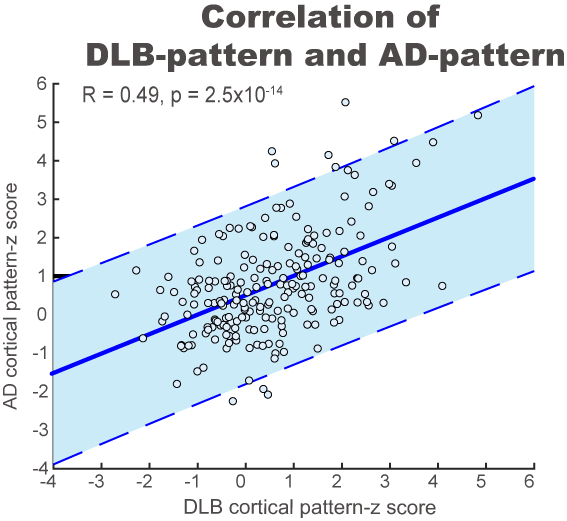
**

**Figure S4. Correlation betweeen the DLB-pattern and the AD-pattern**. Scatter plots of the DLB-pattern and the AD-pattern scores in every participant in this study. The correlation was calculated with Pearson’s correlation analyses.


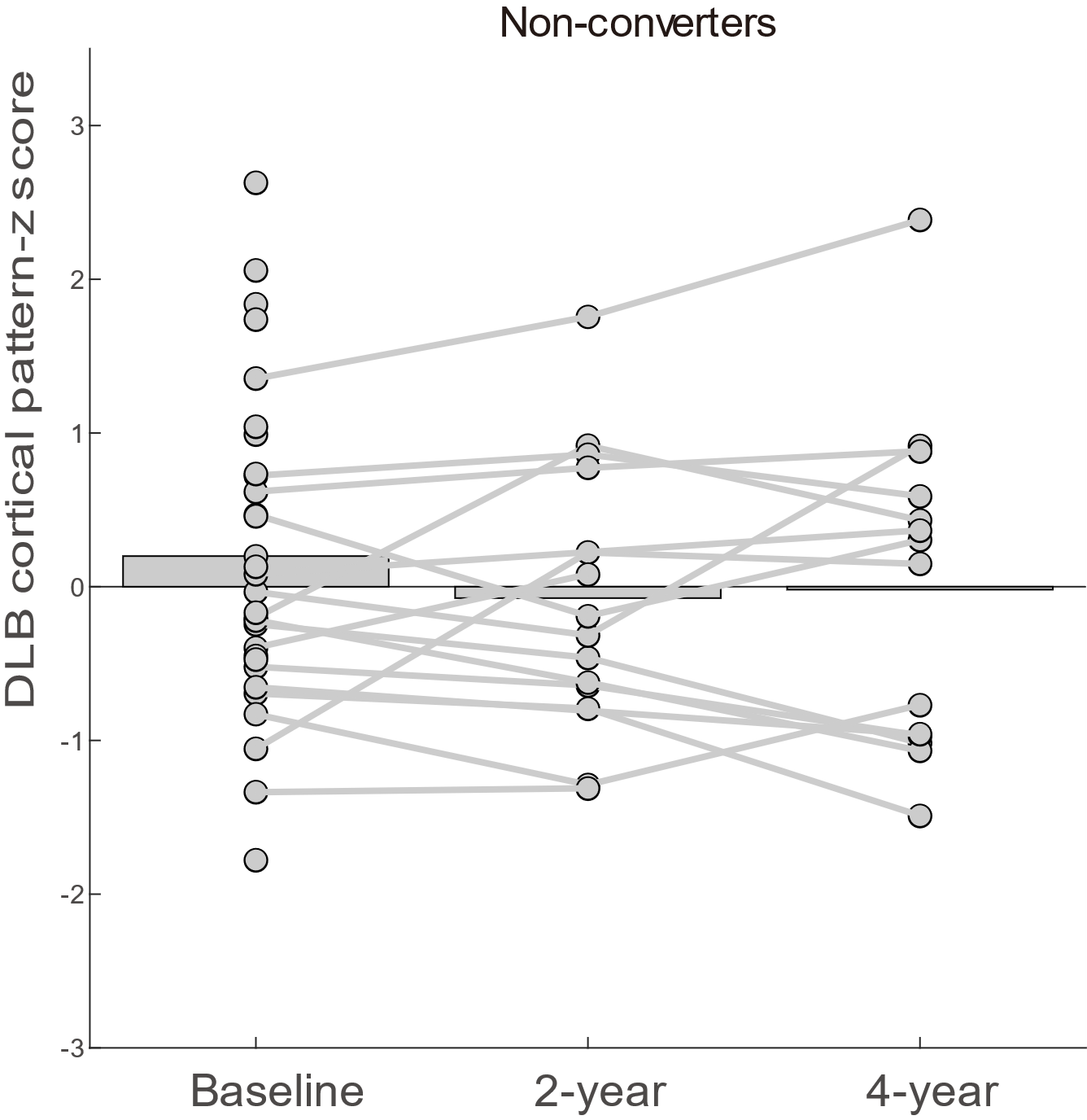


**Figure S5. Longitudinal change of DLB-pattern in the non-converter iRBD group.** Longitudinal change of DLB-pattern from the baseline, 2-years and 4-years follow-up in non-converters.

**Table S1. List of ROIs for cortical thickness analysis**

| 1 | Left_frontomargin | 38 | Left_temporal_middle | 75 | Right_frontomargin | 112 | Right_temporal_middle |
| --- | --- | --- | --- | --- | --- | --- | --- |
| 2 | Left_occipital_inf | 39 | Left_Lat_Fis-ant-Horizont | 76 | Right_occipital_inf | 113 | Right_Lat_Fis-ant-Horizont |
| 3 | Left_paracentral | 40 | Left_Lat_Fis-ant-Vertical | 77 | Right_paracentral | 114 | Right_Lat_Fis-ant-Vertical |
| 4 | Left_subcentral | 41 | Left_Lat_Fis-post | 78 | Right_subcentral | 115 | Right_Lat_Fis-post |
| 5 | Left_transv_frontopol | 42 | Left_Pole_occipital | 79 | Right_transv_frontopol | 116 | Right_Pole_occipital |
| 6 | Left_cingul-Ant | 43 | Left_Pole_temporal | 80 | Right_cingul-Ant | 117 | Right_Pole_temporal |
| 7 | Left_cingul-Mid-Ant | 44 | Left_calcarine | 81 | Right_cingul-Mid-Ant | 118 | Right_calcarine |
| 8 | Left_cingul-Mid-Post | 45 | Left_central | 82 | Right_cingul-Mid-Post | 119 | Right_central |
| 9 | Left_cingul-Post-dorsal | 46 | Left_cingul-Marginalis | 83 | Right_cingul-Post-dorsal | 120 | Right_cingul-Marginalis |
| 10 | Left_cingul-Post-ventral | 47 | Left_circular_insula_ant | 84 | Right_cingul-Post-ventral | 121 | Right_circular_insula_ant |
| 11 | Left_cuneus | 48 | Left_circular_insula_inf | 85 | Right_cuneus | 122 | Right_circular_insula_inf |
| 12 | Left_front_inf-Opercular | 49 | Left_circular_insula_sup | 86 | Right_front_inf-Opercular | 123 | Right_circular_insula_sup |
| 13 | Left_front_inf-Orbital | 50 | Left_collat_transv_ant | 87 | Right_front_inf-Orbital | 124 | Right_collat_transv_ant |
| 14 | Left_front_inf-Triangul | 51 | Left_collat_transv_post | 88 | Right_front_inf-Triangul | 125 | Right_collat_transv_post |
| 15 | Left_front_middle | 52 | Left_front_inf | 89 | Right_front_middle | 126 | Right_front_inf |
| 16 | Left_front_sup | 53 | Left_front_middle | 90 | Right_front_sup | 127 | Right_front_middle |
| 17 | Left_Ins_lcent_ins | 54 | Left_front_sup | 91 | Right_Ins_lcent_ins | 128 | Right_front_sup |
| 18 | Left_insular_short | 55 | Left_interm_prim-Jensen | 92 | Right_insular_short | 129 | Right_interm_prim-Jensen |
| 19 | Left_occipital_middle | 56 | Left_intrapariet_and_P_trans | 93 | Right_occipital_middle | 130 | Right_intrapariet_and_P_trans |
| 20 | Left_occipital_sup | 57 | Left_oc_middle_and_Lunatus | 94 | Right_occipital_sup | 131 | Right_oc_middle_and_Lunatus |
| 21 | Left_oc-temp_lat-fusifor | 58 | Left_oc_sup_and_transversal | 95 | Right_oc-temp_lat-fusifor | 132 | Right_oc_sup_and_transversal |
| 22 | Left_oc-temp_med-Lingual | 59 | Left_occipital_ant | 96 | Right_oc-temp_med-Lingual | 133 | Right_occipital_ant |
| 23 | Left_oc-temp_med-Parahip | 60 | Left_oc-temp_lat | 97 | Right_oc-temp_med-Parahip | 134 | Right_oc-temp_lat |
| 24 | Left_orbital | 61 | Left_oc-temp_med_and_Lingual | 98 | Right_orbital | 135 | Right_oc-temp_med_and_Lingual |
| 25 | Left_pariet_inf-Angular | 62 | Left_orbital_lateral | 99 | Right_pariet_inf-Angular | 136 | Right_orbital_lateral |
| 26 | Left_pariet_inf-Supramar | 63 | Left_orbital_med-olfact | 100 | Right_pariet_inf-Supramar | 137 | Right_orbital_med-olfact |
| 27 | Left_parietal_sup | 64 | Left_orbital-H_Shaped | 101 | Right_parietal_sup | 138 | Right_orbital-H_Shaped |
| 28 | Left_postcentral | 65 | Left_parieto_occipital | 102 | Right_postcentral | 139 | Right_parieto_occipital |
| 29 | Left_precentral | 66 | Left_pericallosal | 103 | Right_precentral | 140 | Right_pericallosal |
| 30 | Left_precuneus | 67 | Left_postcentral | 104 | Right_precuneus | 141 | Right_postcentral |
| 31 | Left_rectus | 68 | Left_precentral-inf-part | 105 | Right_rectus | 142 | Right_precentral-inf-part |
| 32 | Left_subcallosal | 69 | Left_precentral-sup-part | 106 | Right_subcallosal | 143 | Right_precentral-sup-part |
| 33 | Left_temp_sup-T_transv | 70 | Left_suborbital | 107 | Right_temp_sup-T_transv | 144 | Right_suborbital |
| 34 | Left_temp_sup-Lateral | 71 | Left_subparietal | 108 | Right_temp_sup-Lateral | 145 | Right_subparietal |
| 35 | Left_temp_sup-Plan_polar | 72 | Left_temporal_inf | 109 | Right_temp_sup-Plan_polar | 146 | Right_temporal_inf |
| 36 | Left_temp_sup-Plan_tempo | 73 | Left_temporal_sup | 110 | Right_temp_sup-Plan_tempo | 147 | Right_temporal_sup |
| 37 | Left_temporal_inf | 74 | Left_temporal_transverse | 111 | Right_temporal_inf | 148 | Right_temporal_transverse |
